# Prognostic and Clinicopathological Significance of the Systemic Immune-Inflammation Index in Patients With Renal Cell Carcinoma: A Meta-Analysis

**DOI:** 10.3389/fonc.2021.735803

**Published:** 2021-12-07

**Authors:** Mingyu Jin, Shaoying Yuan, Yiming Yuan, Luqi Yi

**Affiliations:** ^1^ Department of Andrology, Guangdong Hospital of Traditional Chinese Medicine, Zhuhai, China; ^2^ Andrology Center, Peking University First Hospital, Beijing, China; ^3^ Department of Urology, Guangdong Hospital of Traditional Chinese Medicine, Zhuhai, China

**Keywords:** systemic immune-inflammation index, prognosis, meta-analysis, renal cell carcinoma, survival

## Abstract

**Background:**

The systemic immune-inflammation index (SII) is a hematological parameter based on neutrophil, platelet, and lymphocyte counts. Studies that have investigated the prognostic value of SII in patients with renal cell carcinoma (RCC) have reported controversial results. In this study, we systematically investigated the prognostic value of SII in patients with RCC.

**Methods:**

We systematically searched English articles in the PubMed, Embase, Web of Science, and Cochrane Library databases up to October 2021. Hazard ratios (HRs) and odds ratios (ORs) with 95% confidence intervals (CIs) were used to obtain pooled results.

**Results:**

The meta-analysis included 10 studies that enrolled 3,180 patients. A high SII was associated with poor overall survival (HR 1.75, 95% CI 1.33–2.30, p<0.001) in patients with RCC. However, a high SII was not shown to be a significant prognostic factor for progression-free survival/disease-free survival (HR 1.22, 95% CI 0.84–1.76, p=0.293) or poor cancer-specific survival (HR 1.46, 95% CI 0.68–3.12, p=0.332) in patients with RCC. A high SII was correlated with male sex (OR 1.51, 95% CI 1.11–2.04, p=0.008), Fuhrman grade G3–G4 (OR 1.80, 95% CI 1.08–3.00, p=0.024), and poor risk based on the International Metastatic Renal Cell Carcinoma Database Consortium criteria (OR 19.12, 95% CI 9.13–40.06, p<0.001).

**Conclusion:**

A high SII was independently associated with poor survival outcomes in patients with RCC. Additionally, an elevated SII indicated more aggressive disease. The SII may serve as a useful cost-effective prognostic indicator in patients with RCC.

## Introduction

Renal cell carcinoma (RCC) is the third most common cancer of the urinary system and accounts for 2.2% of all human malignancies (1). Approximately 25%–30% of patients with RCC present with metastases at the time of diagnosis (2). Among patients diagnosed with early-stage and localized disease, 25% develop recurrence or metastasis after radical surgical resection (3). Immune checkpoint inhibitors are widely accepted as an essential component of RCC treatment following rapid advances in immunotherapy for the management of RCC (4, 5). The prognosis of patients with RCC remains poor; the 5-year survival rate is only 12% for stage IV metastatic disease (6). Prognostic markers are clinically useful for improved management of patients with RCC. Therefore, identification of novel and reliable prognostic indicators is urgently required to improve survival of patients with RCC (7).

The role of the immune system in various stages of cancer progression has been extensively investigated over the last few years (8). Inflammation-based prognostic scores such as platelet-to-lymphocyte ratio (9), lymphocyte to monocyte ratio (10), and prognostic nutritional index (11) are cost-effective and reliable prognostic tools that are widely used in patients with cancer (10, 12). Many studies have shown that the systemic immune-inflammation index (SII) is a useful prognostic marker for several malignant tumors, including pancreatic (13), gallbladder (14), non-small-cell lung (15), and laryngeal cancer (16), as well as for cholangiocarcinoma (17). Studies have investigated the prognostic value of SII in patients with RCC; however, the results are inconsistent (18–25). Therefore, in this meta-analysis, we investigated the role of SII as a prognostic indicator of RCC and also the correlation between SII and clinicopathological features of RCC.

## Materials and Methods

### Study Guideline and Ethics Statement

This meta-analysis was performed in accordance with the Preferred Reporting Items for Systematic Reviews and Meta-Analyses guidelines (26). All data used in this meta-analysis were based on previous studies; therefore, ethical approval and patient consent were not required for this study.

### Search Strategy

The English databases of PubMed, Embase, Web of Science, and Cochrane Library were systematically searched up to October 2021. We used the following search terms: systemic immune-inflammation index OR SII AND renal cell carcinoma OR kidney cancer AND prognosis OR survival OR outcomes OR prognostic. The citation lists of the relevant studies were also manually checked for additional eligible articles. We selected only English publications.

### Inclusion and Exclusion Criteria

The inclusion criteria were as follows: (1) studies that investigated the association between the SII and prognosis in patients diagnosed with RCC, (2) availability of hazard ratios (HRs) and 95% confidence intervals (CIs) for survival outcomes or data required to calculate these values, (3) an appropriately defined SII based on the following formula: platelet count × neutrophil count/lymphocyte count, (4) availability of a cutoff value to divide the SII into high or low SII groups and, (5) articles published in English. The exclusion criteria were as follows: (1) case reports, reviews, meeting abstracts, letters, and comments, (2) duplicate articles with patient overlap, (3) insufficient data for detailed analysis and, (4) animal studies. The survival endpoints included overall survival (OS), progression-free survival (PFS), disease-free survival (DFS), and cancer-specific survival (CSS).

### Data Extraction and Quality Assessment

Two investigators (M.J. and S.Y.) independently extracted information from all studies included in this meta-analysis, and any disagreements were resolved by discussion with a third investigator (Y.Y.). The following data were extracted: first author, publication year, country, sample size, sex, age, study period, survival outcomes, follow-up, cancer type, treatment methods used, cut-off value of the SII, number of patients with high and low SII scores, and HRs and 95% CIs for OS, PFS, DFS, and CSS. The Newcastle–Ottawa quality assessment scale (NOS) (27) was used to assess the quality of the included studies. The NOS assesses the quality of studies with regard to the following aspects: subject selection, comparability of the subject, and clinical outcomes. The NOS score ranged from 0 to 9, and studies with NOS scores ≥6 were considered high-quality studies.

### Statistical Analysis

Pooled HRs and 95% CIs were calculated to determine the role of the SII as a prognostic marker in patients with RCC. Pooled HR >1 (without 95% CI overlapping 1) indicated that a high SII correlated with poor prognosis. Heterogeneity among studies was assessed using the χ2-based Q test and I^2^ statistics. The I^2^>50% and Ph<0.10 indicated significant heterogeneity, and a random-effects model was used for analysis; a fixed-effects model was used in other cases. Subgroup analyses were performed to confirm the source of heterogeneity. The pooled odds ratios (ORs) and 95% CIs were used to determine the association between SII and clinicopathological factors. Pooled OR>1 (without 95% CI overlapping 1) suggested that a high SII was associated with poor clinicopathological outcomes. Potential publication bias was evaluated using the Begg’s test (28). All data analyses were performed using the Stata 12.0 software (Stata Corp LP, College Station, TX, USA). A P value <0.05 (two-tailed) was considered statistically significant.

## Results

### Study Selection

**Figure 1:**
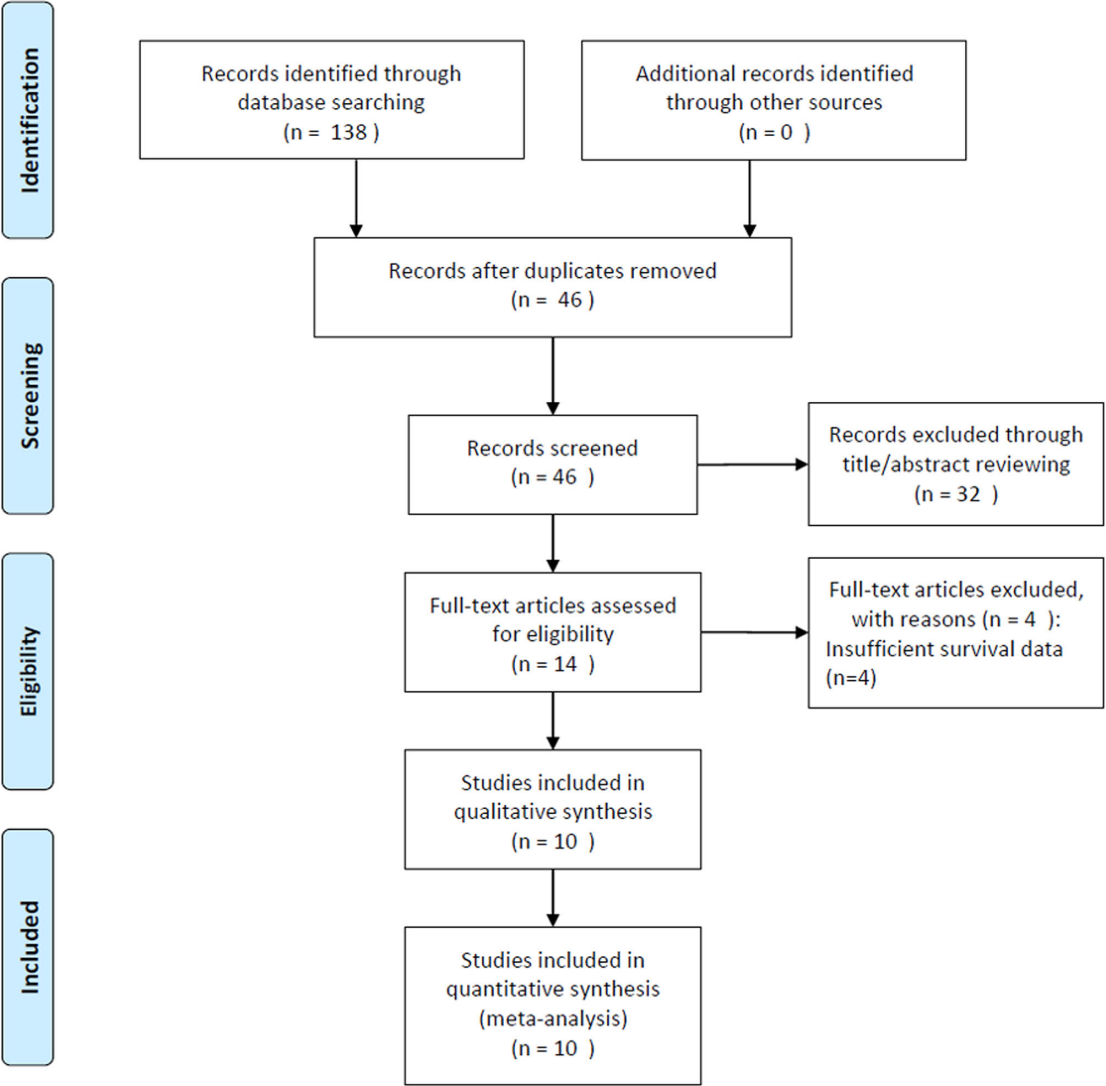
Flow diagram of included studies for this meta-analysis.

**Figure 1** shows a detailed flow diagram of the study selection process. The initial literature search yielded 138 studies, of which 46 were included in the analysis after exclusion of duplicates. After screening of titles and abstracts, 32 studies were discarded and the full text was reviewed in 14. Four studies with insufficient survival data were eliminated. Finally, data of 10 studies that included 3,180 patients (18–25, 29, 30) were analyzed in this meta-analysis.

### Characteristics of Included Studies

**Table 1** summarizes the main characteristics of all studies included in our research. The total sample size was 3,180 and ranged from 31 to 646. Three studies were performed in Turkey (19, 24, 30), two in Italy (21, 23), and one each in India (18), China (22), Japan (25), Austria (29), and Poland (20), respectively. The included studies were published between 2016 and 2021 and all were English publications. All 10 studies investigated the association between SII and OS (18–25, 29, 30), three investigated the association between SII and PFS (18, 23, 30), one between SII and DFS (24), and two between SII and CSS (22, 29). Eight studies recruited patients with metastatic RCC (18–21, 23, 25, 29, 30), and two studies enrolled patients with localized disease (22, 24). The cut-off values of SII ranged from 529 to 1,375 (median 730). All included studies were shown to be high-quality studies (NOS scores ≥6).

**Table 1:** Main characteristics of all included studies.

Author	Year	Country	Sample size	Sex (M/F)	Age (year) Median(range)	Study period	Survival outcome	Follow-up (month)	Cancer type	Treatment methods	Cut-off value	No. of patients with high/low SII	NOS score
Barua	2019	India	31	21/10	Mean: 55	2012-2017	OS, PFS	16.5	mRCC	Surgery	883	17/14	7
Bugdayci	2021	Turkey	187	149/38	61 (34-86)	2012-2019	OS	15	mRCC	Surgery+ TKIs	730	94/93	7
Chrom	2019	Poland	502	339/163	62 (22-88)	2008-2016	OS	52.5	mRCC	TKIs	730	208/294	8
De Giorgi	2019	Italy	313	235/78	65 (40-84)	2015-2016	OS	24	mRCC	ICIs	1375	96/217	7
Hu	2020	China	646	394/252	Mean: 54.77	2010-2013	OS, CSS	84	Localized RCC	Surgery	529	163/483	7
Lolli	2016	Italy	335	238/97	63 (27-88)	2006-2014	OS, PFS	49	mRCC	TKIs	730	126/209	9
Ozbek	2020	Turkey	176	111/65	Mean: 65.32	NR	OS, DFS	NR	Localized RCC	Surgery	830	52/124	6
Teishima	2020	Japan	179	145/34	65.5 (40-85)	2008-2018	OS	24	mRCC	TKIs	730	73/106	8
Laukhtina	2021	Austria	613	NR	65	NR	OS, CSS	31	mRCC	Surgery	710	298/315	7
Yilmaz	2021	Turkey	198	135/63	63 (29–87)	2012-2019	OS, PFS	24(1-70)	mRCC	TKIs	1291	91/107	8

RCC, renal cell carcinoma; mRCC, metastatic renal cell carcinoma; TKIs, tyrosine kinase inhibitors; OS, overall survival; PFS, progression-free survival; DFS, disease-free survival; CSS, cancer-specific survival; ICIs, immune checkpoint inhibitors; NR, not reported; NOS, Newcastle-Ottawa Scale.

### Association Between the Systemic Immune-Inflammation Index and Survival Outcomes in Patients With Renal Cell Carcinoma

**Figure 2:**
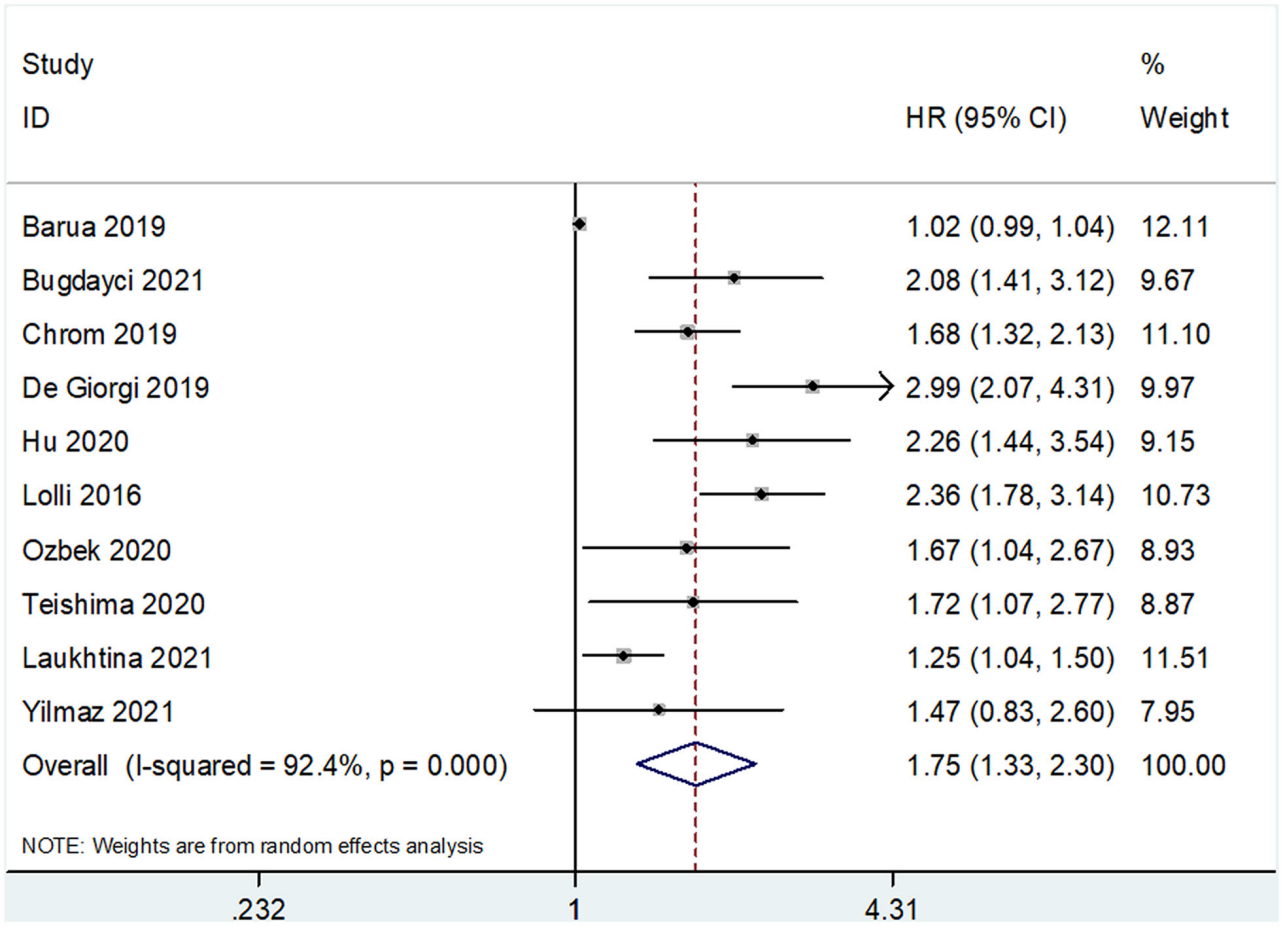
Forest plots showing the association between SII and overall survival (OS) in renal cell carcinoma (RCC).

**Figure 3:**
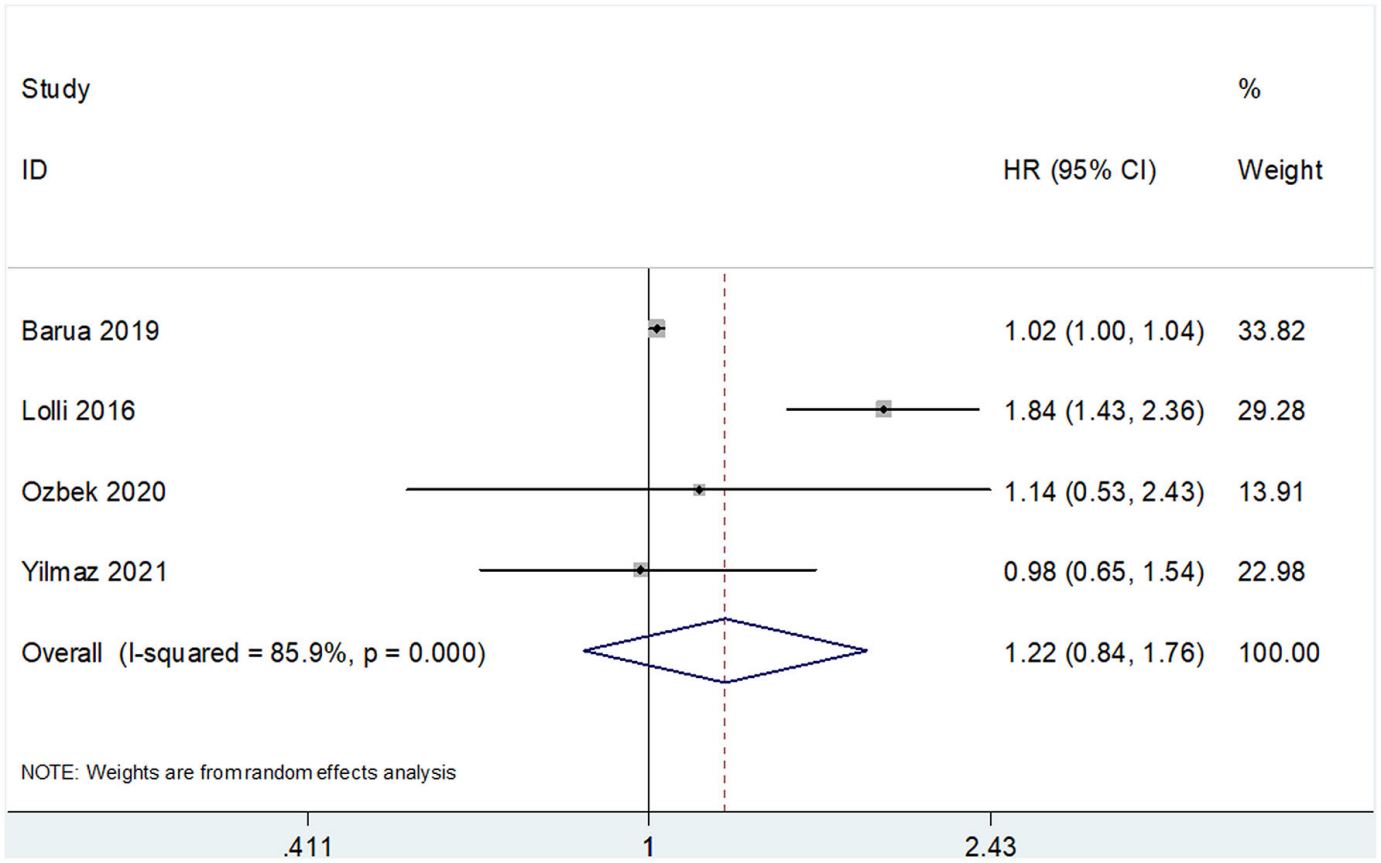
Forest plots showing the association between SII and progression-free survival (PFS)/disease-free survival (DFS) in RCC.

**Figure 4:**
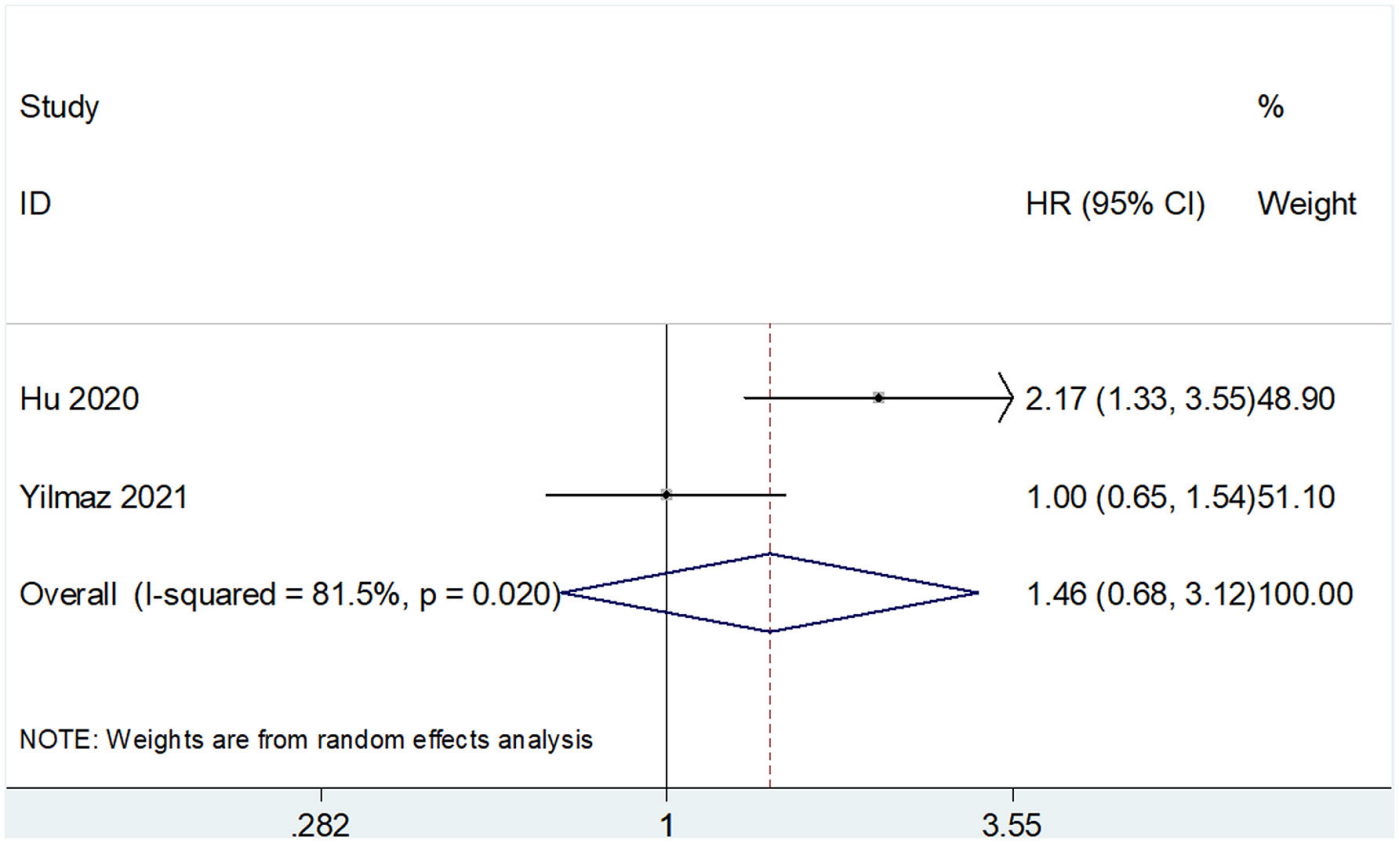
Forest plots showing the association between SII and cancer-specific survival (CSS) in RCC.

The prognostic value of SII for OS was determined based on data from 10 studies that included 3,180 patients (18–25, 29, 30). The pooled HR and 95% CI are as follows: HR 1.75, 95% CI 1.33–2.30, p<0.001 (**Table 2** and **Figure 2**). A random-effects model was used owing to significant heterogeneity (I^2 =^ 92.4%, Ph<0.001). Studies were stratified based on region, cancer type, cut-off value, treatment methods, and sample size for subgroup analyses. A high SII was associated with poor OS, regardless of geographical region, cancer type, and treatment methods (**Table 2**). A high SII was significantly correlated with poor OS at cut-off values ≤730 (HR 1.81, 95% CI 1.41–2.30, p<0.001) (**Table 2**). Four studies that included 740 patients (18, 23, 24, 30) reported an association between SII and PFS/DFS in patients with RCC. Results of pooled data were as follows: HR 1.22, 95% CI 0.84–1.76, p=0.293, which indicate that SII was not a significant prognostic factor for PFS/DFS in patients with RCC (**Table 2** and **Figure 3**). Additionally, subgroup analysis indicated that a cut-off level ≤730 was of prognostic value for poor PFS/DFS in patients with RCC (**Table 2**). Data obtained from two studies (22, 29) showed that a high SII was not associated with poor CSS (pooled data HR 1.46, 95% CI 0.68–3.12, p=0.332) (**Table 2** and **Figure 4**). Subgroup analysis of CSS was not performed because of the limited sample size.

**Table 2:** Subgroup analyses of SII for prognosis in patients with RCC.

Subgroups	No. of studies	No. of patients	Effects model	HR (95%CI)	p	Heterogeneity *I* ^2^(%) Ph	
OS							
Total	10	3,180	Random	1.75 (1.33-2.30)	<0.001	92.4	<0.001
Region							
Asia	6	1,417	Random	1.62 (1.12-2.34)	0.010	85.4	<0.001
Non-Asia	4	1,763	Random	1.92 (1.33-2.78)	0.001	88	<0.001
Cancer type							
Localized RCC	2	822	Fixed	1.96 (1.41-2.71)	<0.001	0	0.363
mRCC	8	2,358	Random	1.70 (1.26-2.30)	0.001	93.2	<0.001
Cut-off value							
≤730	6	2,462	Random	1.81 (1.41-2.30)	<0.001	72.5	0.003
>730	4	718	Random	1.64 (0.92-2.93)	0.096	92.2	<0.001
Treatments							
Surgery	4	1,466	Random	1.37 (1.03-1.81)	0.029	85.5	<0.001
TKIs	4	1,214	Fixed	1.87 (1.58-2.20)	<0.001	27.8	0.245
Surgery + TKIs	1	187	–	2.08 (1.40-3.09)	<0.001	–	–
ICIs	1	313	–	2.99 (2.07-4.31)	<0.001	–	–
Sample size							
≤200	5	771	Random	1.51 (1.04-2.18)	0.029	82.2	<0.001
>200	5	2,409	Random	1.97 (1.42-2.73)	<0.001	85.0	<0.001
PFS/DFS							
Total	4	740	Random	1.22 (0.84-1.76)	0.293	85.9	<0.001
Region							
Asia	3	405	Fixed	1.02 (1.00-1.04)	0.048	0	0.943
Non-Asia	1	335	–	1.84 (1.43-2.36)	<0.001	–	–
Cancer type							
Localized RCC	1	176	–	1.14 (0.53-2.43)	0.738	–	–
mRCC	3	564	Random	1.23 (0.81-1.88)	0.330	90.6	<0.001
Cut-off value							
≤730	1	335	–	1.84 (1.43-2.36)	<0.001	–	–
>730	3	405	Fixed	1.02 (1.00-1.04)	0.048	0	0.943
Treatments							
Surgery	2	207	Fixed	1.02 (1.00-1.04)	0.047	0	0.777
TKIs	2	533	Random	1.38 (0.74-2.55)	0.311	83.6	0.014
Sample size							
≤200	3	405	Fixed	1.02 (1.00-1.04)	0.048	0	0.943
>200	1	335	-0	1.84 (1.43-2.36)	<0.001	–	–
CSS							
Total	2	1,259	Random	1.46 (0.68-3.12)	0.332	81.5	0.020

RCC, renal cell carcinoma; mRCC, metastatic renal cell carcinoma; TKIs, tyrosine kinase inhibitors; OS, overall survival; PFS, progression-free survival; DFS, disease-free survival; CSS, cancer-specific survival; ICIs, immune checkpoint inhibitors.

### Correlation Between the Systemic Immune-Inflammation Index and Clinicopathological Factors in Patients With Renal Cell Carcinoma

**Figure 5:**
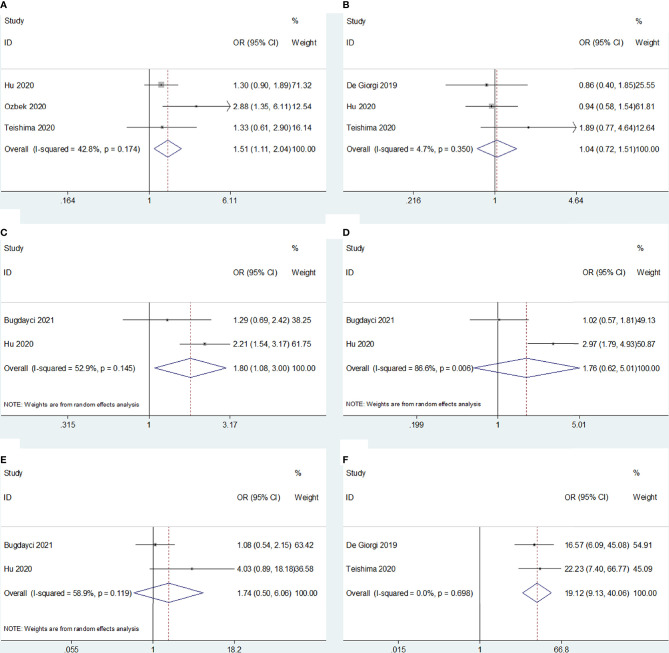
Forest plots of the association between SII and clinicopathological features of RCC. **(A)** Sex; **(B)** Histological type; **(C)** Fuhrman grade; **(D)** T stage; **(E)** Sarcomatoid differentiation, and **(F)** IMDC risk.

Five studies (19, 21, 22, 24, 25) reported an association between SII and clinicopathological characteristics in RCC; sex (male vs. female), histopathological type (clear cell [ccRCC] vs. non-ccRCC), Fuhrman grade (G3–G4 vs. G1–G2), T stage (T3–T4 vs. T1–T2), sarcomatoid differentiation (present vs. absent), and the International Metastatic Renal Cell Carcinoma Database Consortium (IMDC) risk score (poor vs. favorable/intermediate) were associated with SII. The results showed that a high SII was correlated with male sex (OR 1.51, 95% CI 1.11–2.04, p=0.008), Fuhrman grade G3–G4 (OR 1.80, 95% CI 1.08–3.00, p=0.024), and poor risk based on IMDC criteria (OR 19.12, 95% CI 9.13–40.06, p<0.001) (**Figure 5** and **Table 3**). However, we observed no significant association between the SII and histopathological cancer type (OR 1.04, 95% CI 0.72–1.51, p=0.840), T stage (OR 1.76, 95% CI 0.62–5.01, p=0.292), or sarcomatoid differentiation (OR 1.74, 95% CI 0.50–6.06, p=0.382) (**Figure 5** and **Table 3**).

**Table 3:** The meta-analysis of association between SII and clinicopathological factors in patients with RCC.

Variables	No. of studies	No. of patients	Effects model	OR (95%CI)	p	Heterogeneity *I* ^2^(%) Ph	
Sex (male vs female)	3	1,001	Fixed	1.51(1.11-2.04)	0.008	42.8	0.174
Histological type (non-clear cell vs clear cell)	3	1,138	Fixed	1.04(0.72-1.51)	0.840	4.7	0.350
Fuhrman grade (G3-G4 vs G1-G2)	2	833	Random	1.80(1.08-3.00)	0.024	52.9	0.145
T stage (T3-T4 vs T1-T2)	2	833	Random	1.76(0.62-5.01)	0.292	86.6	0.006
Sarcomatoid differentiation (present vs absent)	2	833	Random	1.74(0.50-6.06)	0.382	58.9	0.119
IMDC risk (poor vs favorable/intermediate)	2	492	Fixed	19.12(9.13-40.06)	<0.001	0	0.698

IMDC, International Metastatic Renal Cell Carcinoma Database Consortium.

### Publication Bias

**Figure 6:**
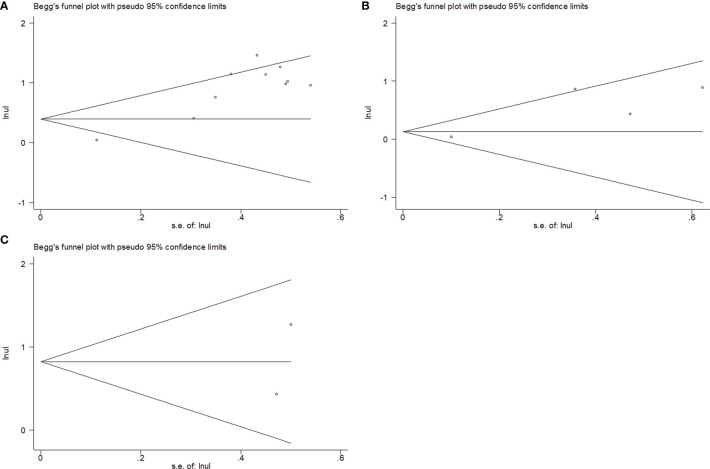
Publication bias assessment using Begg funnel plot. **(A)** Begg’s test for OS; **(B)** Begg’s test for PFS/DFS; **(C)** Begg’s test for CSS.

As shown in **Figure 6**, we observed no significant publication bias in our meta-analysis based on funnel plots and Begg’s test (p=0.592 for OS, p=0.734 for PFS/DFS, and p=1 for CSS).

## Discussion

The SII has been reported as a useful prognostic indicator in many solid tumors, including gallbladder (31), pancreatic (13), and colorectal cancer (32), as well as in intrahepatic cholangiocarcinoma (33). Studies have investigated the association between SII and survival outcomes in patients with RCC (18–25, 29, 30); however, the results remain controversial. In the current meta-analysis, we analyzed data of 10 studies that included 3,180 patients and quantitatively investigated the role of SII as a prognostic indicator in RCC. Pooled data showed that a high SII was associated with poor OS but not with PFS/DFS or CSS in patients with RCC. Furthermore, a high SII was also correlated with a high Fuhrman grade and poor IMDC risk scores. In this meta-analysis, we observed that a high SII indicated poor survival outcomes and aggressive histopathological features in patients with RCC. To our knowledge, this is the first meta-analysis that investigated the prognostic value of the SII in patients with RCC. The immune system plays a critical role in tumor development *via* various mechanisms including tumor initiation, angiogenesis, and metastasis (34). The tumor microenvironment (TME) can trigger immune inflammatory responses and facilitate tumor progression (35). For example, natural killer and CD8+ T cells in the TME can recognize and eliminate more immunogenic cancer cells during the early stages of tumor development (36). Moreover, M2-type tumor-associated macrophages are protumorigenic and promote angiogenesis, lymphangiogenesis, and cancer cell proliferation and metastasis in the TME (37).

The SII, calculated using blood test parameters, is a useful prognostic indicator based on the following underlying mechanisms: (a) neutrophils participate in different stages of tumor progression *via* production of a variety of cytokines (38). Neutrophils in the TME release various cytokines and chemokines such as reactive oxygen species and transforming growth factor (TGF)-β to educate themselves and other cell types to differentiate into a pro-cancer phenotype (39, 40). (b) Platelets stimulate thrombopoiesis and tumor angiogenesis *via* production of TGF-β, promotion of adhesion, and prevention of cell death (41). (c) Cytotoxic lymphocytes play an important role in the cell-mediated immunological destruction of tumor cells (42). Lymphocytosis represents activation of the immune response and is associated with prolonged survival in patients with cancer (43). Therefore, a high SII, which could be secondary to elevated neutrophil or platelet counts, and/or low lymphocyte counts, is correlated with poor survival outcomes in patients with RCC. Notably, our results also indicate that a high SII was associated with a high Fuhrman grade and poor IMDC risk scores. The Fuhrman grade and IMDC risk scores reflect aggressiveness of the cancer; therefore, patients with a high SII tend to show tumor progression or recurrence after initial treatment.

Recent meta-analyses have investigated the prognostic role of SII in many cancer types, including hepatocellular (44), gastric (45), breast (46), and colorectal cancer (47). A meta-analysis that included 2,796 patients reported that a high SII was associated with poor prognosis in patients with hepatocellular carcinoma (44). Fu et al. observed that a high SII was significantly associated with poor OS and DFS in patients with gastric cancer (45). Huang et al. also reported that a high SII was associated with poor OS, PFS, and CSS in patients with urologic cancers (48). A recent meta-analysis observed that a high SII predicts poor survival outcomes in patients with gynecological cancers (49). The results of the aforementioned meta-analyses are consistent with our findings. Moreover, we observed an association between the SII and Fuhrman grade and IMDC risk scores in patients with RCC, which highlights the clinical usefulness of the SII to identify patients at high risk of tumor progression.

In a recent study, the authors performed transcriptome profiling of all three subgroups of RCC using machine learning and bioinformatics analysis (50); transcriptomic data of 891 patients were extracted from The Cancer Genome Atlas (TCGA) database; ccRCC samples obtained from mixed subgroups showed an inverse correlation between mitochondrial and angiogenesis-related genes in the TCGA database and external validation cohorts (50). Moreover, affiliation to the mixed subgroup was associated with a significantly shorter OS in patients with ccRCC and longer OS in patients with chromophobe RCC (50). These findings reported by Marquardt et al. (50) indicate heterogeneity among various histopathological subtypes of RCC, which can be attributed to the different gene clusters in each subgroup. These findings highlight the heterogeneity among recruited patients because the histopathological types were not the same.

Following are the limitations of this meta-analysis: (i) The relatively small sample size is a drawback of this research; this meta-analysis included only 10 studies that investigated 3,180 patients. Large-scale studies are warranted in future to provide deeper insight into this subject. (ii) The cut-off values of SII varied across the included studies, which may have contributed to a selection bias. (iii) Most studies were retrospectively designed; therefore, the inherent flaws associated with retrospective studies may have introduced heterogeneity in the meta-analysis, although we did not detect publication bias.

## Conclusions

This meta-analysis highlights that a high SII was independently associated with poor survival outcomes in patients with RCC. Additionally, a high SII indicates greater aggressiveness of the malignancy. The SII may serve as a useful cost-effective prognostic indicator in patients with RCC.

## Data Availability Statement

The original contributions presented in the study are included in the article/supplementary material. Further inquiries can be directed to the corresponding author.

## Author Contributions

MJ and SY provided the study conception and design. YY and LY contributed to the drafting of the article and final approval of the submitted version. All authors provided the analyses and interpretation of the data and completion of figures and tables. All authors contributed to the article and approved the submitted version.

## Conflict of Interest

The authors declare that the research was conducted in the absence of any commercial or financial relationships that could be construed as a potential conflict of interest.

## Publisher’s Note

All claims expressed in this article are solely those of the authors and do not necessarily represent those of their affiliated organizations, or those of the publisher, the editors and the reviewers. Any product that may be evaluated in this article, or claim that may be made by its manufacturer, is not guaranteed or endorsed by the publisher.
